# Secretor status-dependent modulation of *in vitro* immune responses by human milk oligosaccharides

**DOI:** 10.3389/fnut.2026.1791158

**Published:** 2026-06-12

**Authors:** Marit Zuurveld, Anneke Hellinga, Kennedy Spann, Lars Bode, Belinda van’t Land, Johan Garssen

**Affiliations:** 1 Division of Pharmacology, Utrecht Institute for Pharmaceutical Sciences (UIPS), Utrecht University, Utrecht, Netherlands; 2 Center for Translational Immunology, University Medical Center Utrecht, Utrecht, Netherlands; 3 Department of Pediatrics, Larsson-Rosenquist Foundation Mother-Milk-Infant Center of Research Excellence (MOMI CORE), Human Milk Institute (HMI), University of California San Diego, La Jolla, CA, United States; 4 Danone Research & Innovation Center, Utrecht, Netherlands

**Keywords:** breastfeeding, early life immunity, FUT2 expression, human milk oligosaccharides, mucosal immunity, Secretor status

## Abstract

**Introduction:**

Human milk oligosaccharides (HMOs) are structurally diverse carbohydrates found in high concentrations in human milk, supporting infant immune development and gut microbiota colonization. Their composition is influenced by maternal Secretor (Se) status, determined by the FUT2 gene. While immunological HMO effects are increasingly recognized, the influence of an infant’s own Se status on immune responses remains understudied. This study explores how peripheral blood mononuclear cells (PBMCs) from Se-positive (Se+) and Se-negative (Se−) individuals respond to Se+ and Se− HMOs under bacterial and viral stimulation.

**Methods:**

PBMCs from 14 healthy adult donors, classified based on FUT2 expression, were exposed to 0.1% pooled Se+ or Se− HMOs, individual HMOs (2′-fucosyllactose and 3-fucosyllactose), and immune triggers (αCD3/CD28, LPS, and/or Poly I:C) for 24–48 h. Cytokine secretion (IFNγ, IL10, IL13, TNFα) was measured.

**Results:**

Basal cytokine secretion did not differ between Se+ and Se− PBMCs. Upon stimulation, Se+ PBMCs secreted more IL10, particularly in response to Se+ or Se− HMOs. Individual HMOs did not replicate effects seen with pooled mixtures, highlighting the importance of HMO complexity. Under LPS stimulation, TNFα secretion was significantly reduced only with genotype-matched HMOs, suggesting Secretor-specific immune modulation.

**Conclusion:**

This is the first study showing that PBMC cytokine responses are shaped mainly by host Secretor status than HMO composition. Both genotype and HMO profile influence immune reactivity and should be considered in HMO research and personalized infant nutrition strategies.

## 1 Introduction

Human milk oligosaccharides (HMOs) are bioactive carbohydrates that constitute the third most abundant solid component in human milk, following lactose and lipids (1). These molecules play a pivotal role in shaping the infants’ gut microbiota and modulating immune system development (2). HMOs exert a range of beneficial effects, including the promotion of beneficial bacterial growth, inhibition of pathogen adhesion, and modulation of immune responses (3). The HMO composition is particularly influenced by the Secretor status of the mother, which is determined by the expression of a functional *fucosyltransferase 2* (*FUT2*) gene. Mothers with an active FUT2 gene produce HMOs enriched in specific α1,2-fucosylated structures, whereas mothers with an inactive *FUT2* gene lack these fucosylated motifs, resulting in a distinct HMO profile of over 100 different structures (4).

The widespread expression of FUT2 in secretory tissues suggests that *FUT2* plays a significant role in modulating immune responses during host microbial interaction, possibly through the secretion of fucosylated glycans (5, 6). These FUT2-mediated glycosylation patterns contribute to mucosal barrier integrity, immune cell recruitment, cytokine signaling, and pathogen recognition (5, 7). Preclinical studies demonstrate that upregulation of *FUT2* expression is associated with protection against the development of necrotizing enterocolitis (8). On the other hand, individuals with an active *FUT2* gene (Secretors; Se+) are more susceptible to intestinal viral infections such as norovirus and rotavirus than individuals with an inactive *FUT2* gene (non-Secretors; Se−) (9–11). It is important to note that *FUT2* expression is mostly categorized in binary terms—either present or absent—yet recent genomic and glycomic analyses reveal that its expression exists along a continuum, influenced by both genetic variation and regulatory mechanisms. This gradient of expression contributes to a spectrum of HMO profiles, which may have differential effects on infant health, including susceptibility to respiratory conditions such as recurrent wheeze (12).

Recent research has highlighted the direct immunomodulatory effects of HMOs. HMOs interact with immune cell receptors, modulate cytokine production, and influence the maturation of immune cells such as dendritic cells and T cells (13–15). Specific HMOs, such as 2′-fucosyllactose (2’FL) and 3-fucosyllactose (3FL), have been shown to modulate inflammation and allergic responses in both *in vitro* and *in vivo* preclinical research models (16, 17). Although 2’FL and 3FL are the most characteristic HMOs found in Se+ and Se− HMOs profiles, they do not represent the potential immunomodulatory effects of the full HMOs compositions. Recently, we demonstrated that pooled Se+ and Se− HMOs differentially modulate immune responses in an *in vitro* sensitization model using the cow’s milk allergen *β*-lactoglobulin. Se+ HMOs promoted a type 2 cytokine profile and increased regulatory T cell induction, while Se− HMOs dampened dendritic cell activation and did not elicit a type 2 response (14). These findings underscore that Se+ and Se− HMOs are not functionally equivalent, potentially resulting in different clinical outcomes.

While most studies so far have focused on maternal Secretor status and human milk composition with regards to infant development and health (18), our understanding of how infants’ Secretor status influences its development and health remains unknown. Emerging evidence suggests that a match or mismatch between maternal and infant Secretor status may influence immune outcomes (19), potentially through differences in HMO composition and host–microbe interactions. Our study addresses this gap by comparing *in vitro* immune responses of peripheral blood mononuclear cells (PBMCs) from Se+ and Se− individuals when exposed to Se+ or Se− HMO profiles as well as bacterial and viral immune triggers. By studying functional immune responses under these conditions, we aim to gain insights into the potential immunomodulatory effects of HMOs and their relevance to infant Secretor status.

## 2 Materials and methods

### 2.1 Human milk oligosaccharides

Human milk was collected as part of UC San Diego’s Human Milk Institute human milk donation program. Milk was screened by fluorescence high-performance liquid chromatography (HPLC-FL) to determine Secretor status. HMOs were isolated as previously described (20). HMOs were dissolved in PBS and purified from LPS. Further details on milk collection, HMOs isolation and purification are described by Hellinga et al. (14).

Lyophilized enzymatically (lactose-derived) produced 2′FL and 3FL (Carbosynth, UK) were dissolved in sterile PBS and stored in −20 °C until further use.

### 2.2 PBMC isolation

Human PBMCs were isolated from buffy coats from healthy donors (Dutch National Blood Bank, The Netherlands). Cells were collected into an enriched fraction by density gradient centrifugation (1,000 g, 13 min) using Leucosep tubes (Greiner Bio-One, Austria). The enriched cell fraction was washed three times with 45 mL PBS containing 2% FCS. Erythrocytes were lysed by incubation for 5 min in red blood cell lysis buffer (4.14 g NH_4_CL, 0.5 g KHCO_3_, 18.6 mg Na_2_EDTA in 500 mL demi water, sterile filtered, pH = 7.14) on ice. PBMCs were resuspended in RPMI 1640 (Gibco, USA) supplemented with 2.5% FCS, penicillin (100 U/mL) and streptomycin (100 μg/mL) for counting. PBMCs were stored in 90% FCS + 10% DMSO in liquid nitrogen until further use.

### 2.3 PBMC stimulation

**Figure 1:**
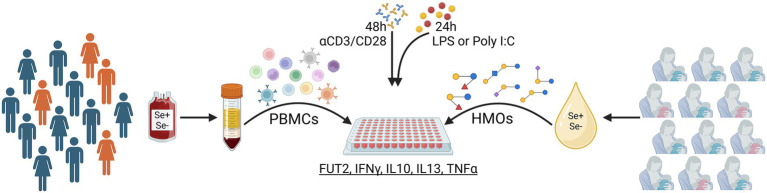
Schematic overview of experimental design. In short, PBMCs from 14 healthy volunteers were used to determine FUT2 enzyme expression. PBMCs were incubated with Se+ HMOS, Se− HMOS, 2’FL, or 3FL and were either unstimulated or stimulated with αCD3/CD28 for 48 h. An additional stimulation of LPS or Poly I:C was provided in the final 24 h of the incubation. Secreted cytokine levels were determined to investigate immune responses. Created with Biorender.com.

After thawing, PBMCs (2×10^5 cells per condition) were incubated in 200 μL RPMI containing 2.5% FCS, penicillin (100 U/mL) and streptomycin (100 μg/mL) in a 96 wells plate. PBMCs were incubated with 0.1% HMOs and some conditions also with 150 ng/mL αCD3/CD28 (BD Biosciences, USA) for 48 h. During the final 24 h of the HMOs incubation, some wells were stimulated with 100 ng/mL LPS (Invivogen, USA) or 100 μg/mL Poly I:C (Invivogen, USA), in addition to the already present αCD3/CD28. Figure 1 provides a schematic overview of the experimental design used in this study.

After 48 h incubation cells were partially collected for viability assessment (data not shown) and partially collection for lysing in RIPA buffer (Thermo Scientific, USA) containing protease inhibitors (Roche, Switzerland). Cell lysates were stored at −20 °C to measure FUT2 protein levels. Supernatants were collected and stored to measure secreted cytokines.

### 2.4 FUT2 protein expression and cytokine measurement

Stored unstimulated cell lysates were used to determine the presence of FUT2 protein. A FUT2 ELISA kit (MyBioSource, USA) was used according to manufacturer’s instructions.

Secreted levels of IFNγ, IL10, IL13 and TNFα in cell culture supernatants were determined by ELISA (Thermo Fisher Scientific, USA) according to manufacturer’s instructions.

### 2.5 Statistical analysis

Normal distribution of data was tested prior to further statistical analysis. When data did not fit a normal distribution, square root or logarithmic transformations were performed. Differences between stimulations with control were tested for statistical significance by One-Way ANOVA followed by Dunnett’s posthoc test or Two-Way ANOVA followed by Sidak’s *post hoc* test. Pearson correlations with simple linear regressions were used to test for correlations between FUT2 expression and cytokine secretions. *p*-values <0.05 were considered statistically significant. Data is presented as mean ± SEM for 14 independent biological donors. Graphpad Prism 10 software was used.

## 3 Results

### 3.1 PBMCs from Se+ donors have a higher cytokine secretion capacity than PBMCs from Se− donors in response to different immune triggers

**Figure 2:**
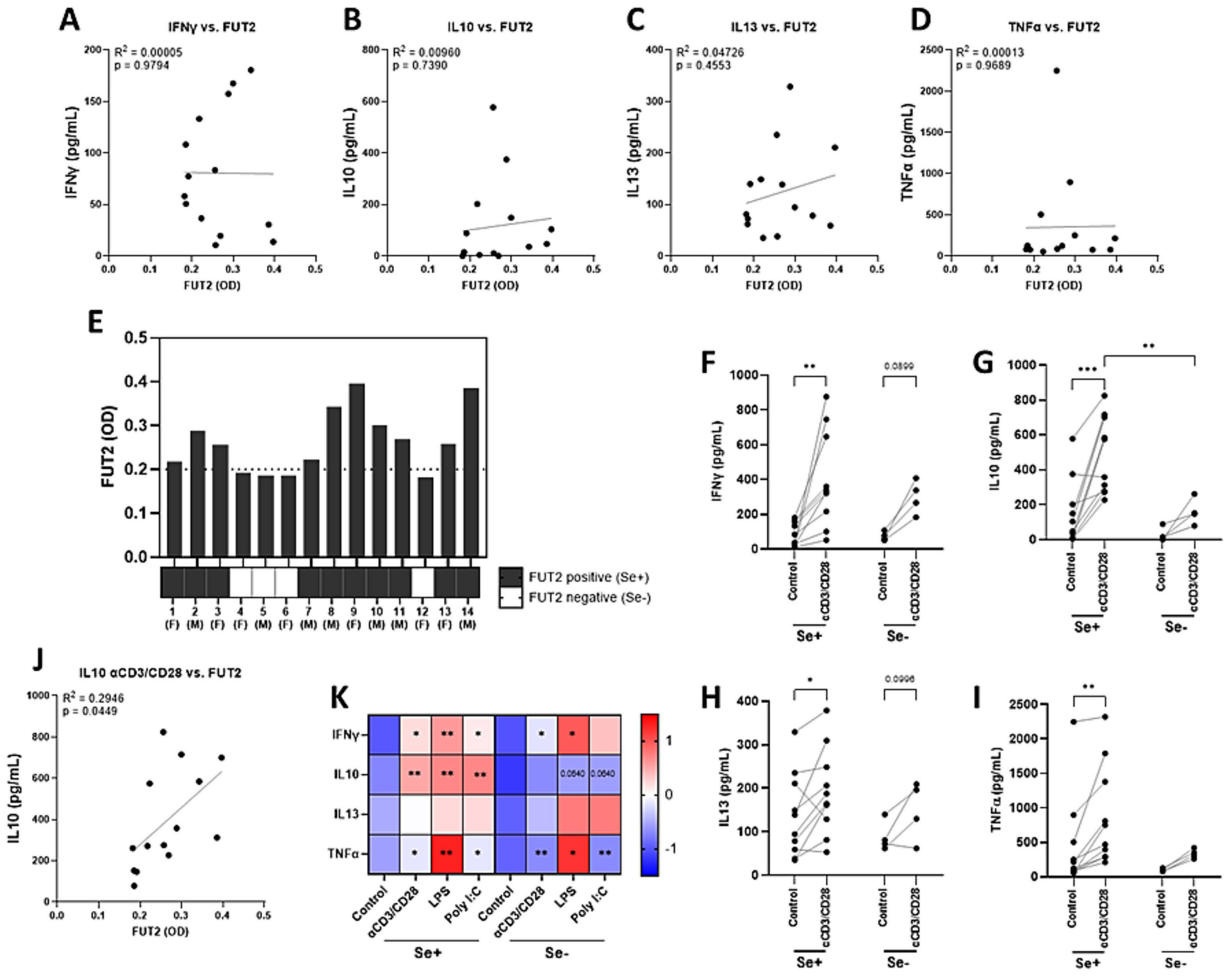
PBMC cytokine secretion in relation to fucosyltransferase-2 (FUT2) protein expression. PBMCs of 14 independent healthy donors were cultured for 48 h to determine cytokine secretion and presence of intracellular FUT2 protein. Secretion of **(A)** IFNγ, **(B)** IL10, **(C)** IL13, **(D)** TNFα during 48 h in unstimulated PBMCs in correlation to FUT2 protein expression in PBMC lysates were assessed. **(E)** The FUT2 protein expression by unstimulated PBMCs was used to determine Secretor status of PBMC donors (Se+ or Se−). Secretion of cytokines **(F)** IFNγ, **(G)** IL10 **(H)** IL13, and **(I)** TNFα were measured upon 48 h αCD3/CD28 stimulation of Se+ and Se− PBMCs. **(J)** Correlation between IL10 secretion after 48 h αCD3/CD28 stimulation of PBMCs and FUT2 protein expression. **(K)** *Z*-scores were calculated of cytokine levels for both Se+ and Se− donors after αCD3/CD28, LPS, or Poly I:C stimulation. Data (*n* = 14 healthy PBMC donors) is analyzed by Pearson correlation test and a simple linear regression. Increases in cytokine secretion upon stimulation of PBMCs were analyzed by Two-Way ANOVA followed by Bonferroni *post hoc* test and displayed as mean ± SEM (*p < 0.05; **p < 0.0.1).

Upon incubation of PBMCs for 48 h, FUT2 protein expression and secreted cytokines were measured to determine the Secretor status. First an unbiased approach between FUT2 expression and cytokine levels was used in unstimulated cells. No significant correlations were found between the level of FUT2 expression and cytokine secretion (Figures 2A–D). To distinguish Se+ and Se− PBMC donors based on FUT2 protein expression, a blank control (RPMI medium only) was used as cut-off value to separate the donors in Se+ and Se− groups (Figure 2E).

Comparing αCD3/CD28 stimulated with unstimulated (Control) cells within and between Se+ and Se− donors, a significant increase in IFNγ, IL10, IL13 and TNFα release was observed within Se+ donors (Figures 2F–I). On the other hand, in Se− donors production of IFNγ and IL13 s tended to be increased upon αCD3/CD28 stimulation (*p* = 0.0899 and *p* = 0.996 respectively). Secretion levels of IL10 in Se+ donors upon αCD3/CD28 stimulation was significantly higher than the observed secretion IL10 in Se− donors upon αCD3/CD28 stimulation (*p* = 0.0056, Figure 2G).

To analyze the relation between FUT2 gene expression and cytokine secretion upon stimulation we performed a Spearman correlation analysis. A significant positive correlation (R^2^ = 0.2946, *p* = 0.0449) was found between FUT2 protein expression and IL10 levels after αCD3/CD28 stimulation (Figure 2J), which was not found for any of the other measured cytokines (Supplementary Figures 1A–C). Apart from αCD3/CD28 stimulation, additional LPS or Poly I: C stimulations also demonstrated that the Se+ donors have an increased capacity of significantly upregulated IL10 secretion in response to these stimuli (Figure 2K; Supplementary Figures E–H).

### 3.2 PBMCs from Se+ donors exhibit a higher capacity for IL10 secretion, while exposure to all HMOs results in a reduced IL13 secretion in PBMCs from Se− donors

PBMCs of Se+ and Se− donors were incubated for 48 h in the presence of 0.1% Se+ HMOs, Se− HMOs, 2’FL or 3FL and subsequently cytokine secretion was determined.

**Figure 3:**
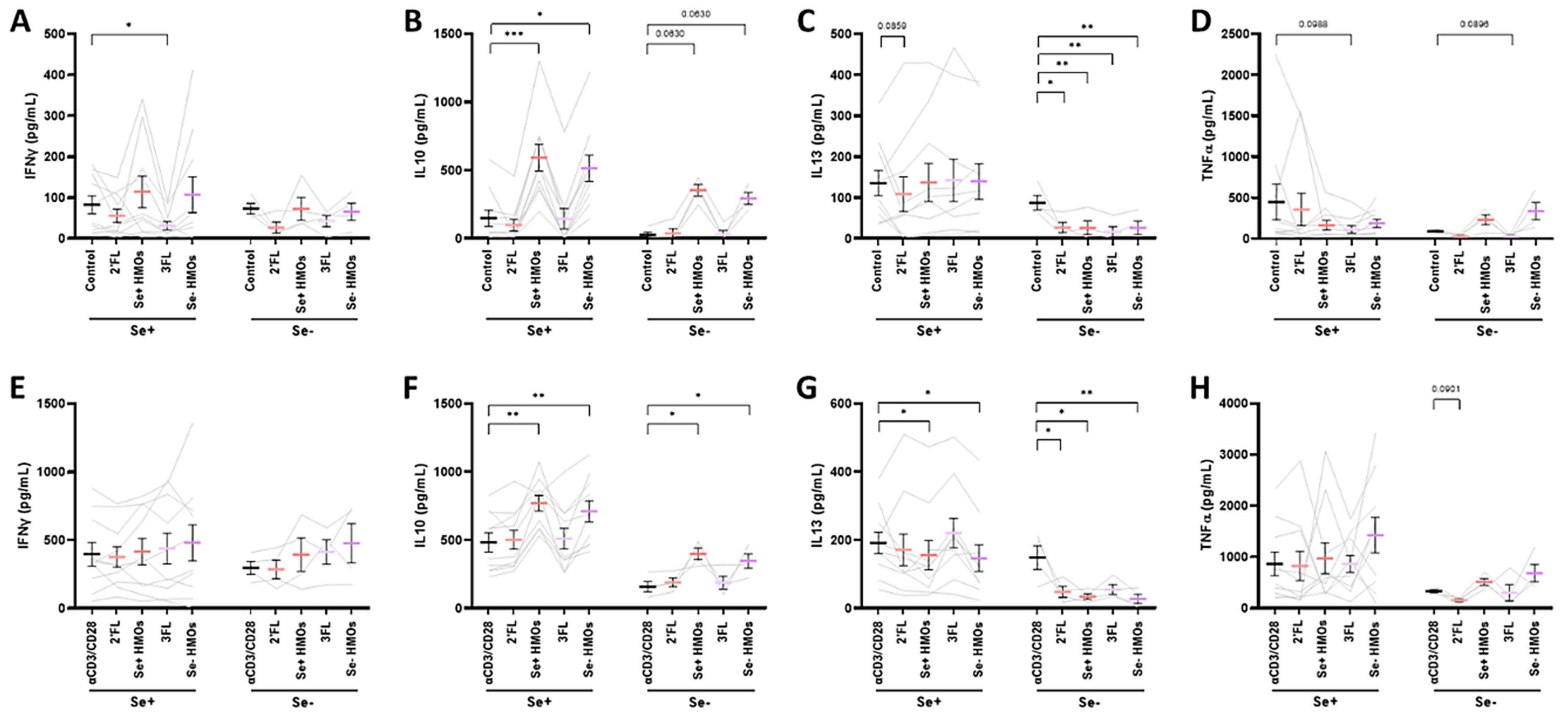
Immunomodulatory effects of single and pooled Se+ or Se− HMOs on cytokine secretion in unstimulated **(A–D)** and αCD3/CD28 stimulated **(E–H)** Se+ and Se− PBMCs. PBMCs of 14 independent healthy donors were cultured for 48 h (with or without αCD3/CD28 stimulation) in the presence of 0.1 w/v% 2’FL, Se+ HMOs, 3FL or Se− HMOs to determine secretion of **(A,E)** IFNγ, **(B,F)** IL10, **(C,G)** IL13, and **(D,H)** TNFα. Data (*n* = 14 healthy PBMC donors) is analyzed by One-Way ANOVA and Dunnett’s post hoc test. Data is displayed as mean ± SEM (*p < 005; **p < 0.01; ***p < 0.001).

When Se+ PBMCs were incubated with 3FL, a significantly lower IFNγ secretion was observed compared to the control condition (Figure 3A). IL10 secretion was significantly increased when Se+ PBMCs were incubated with either Se+ HMOs or Se− HMOs, but not with 2’FL or 3FL (Figure 3B). Se− PBMCs only showed a tendency to increased IL10 secretion upon exposure to Se+ HMOs or Se− HMOS compared to control (*p* = 0.0630). No significant changes were observed for IL13 secretion in Se+ PBMCs following incubation with HMOs. In contrast, IL13 levels decreased significantly across all HMOs conditions in Se− PBMCs (Figure 3C). While no significant differences were observed in TNFα responses, secretion upon 3FL incubation tended to be lower in both Se+ and Se− PBMCs as compared to the control (*p* = 0.0988 and *p* = 0.0896 respectively, Figure 3D).

Most effects on cytokine secretion in response to HMOs remained similar when αCD3/CD28 was added during HMOs incubation (Figures 3E,F,H). However, decreased IL13 secretion was observed in Se+ PBMCs incubated with Se+ or Se− HMOs combined with αCD3/CD28 stimulation (Figure 3G).

### 3.3 Degree of LPS and Poly I:C induced immune responses depends on PBMC Secretor status

**Figure 4:**
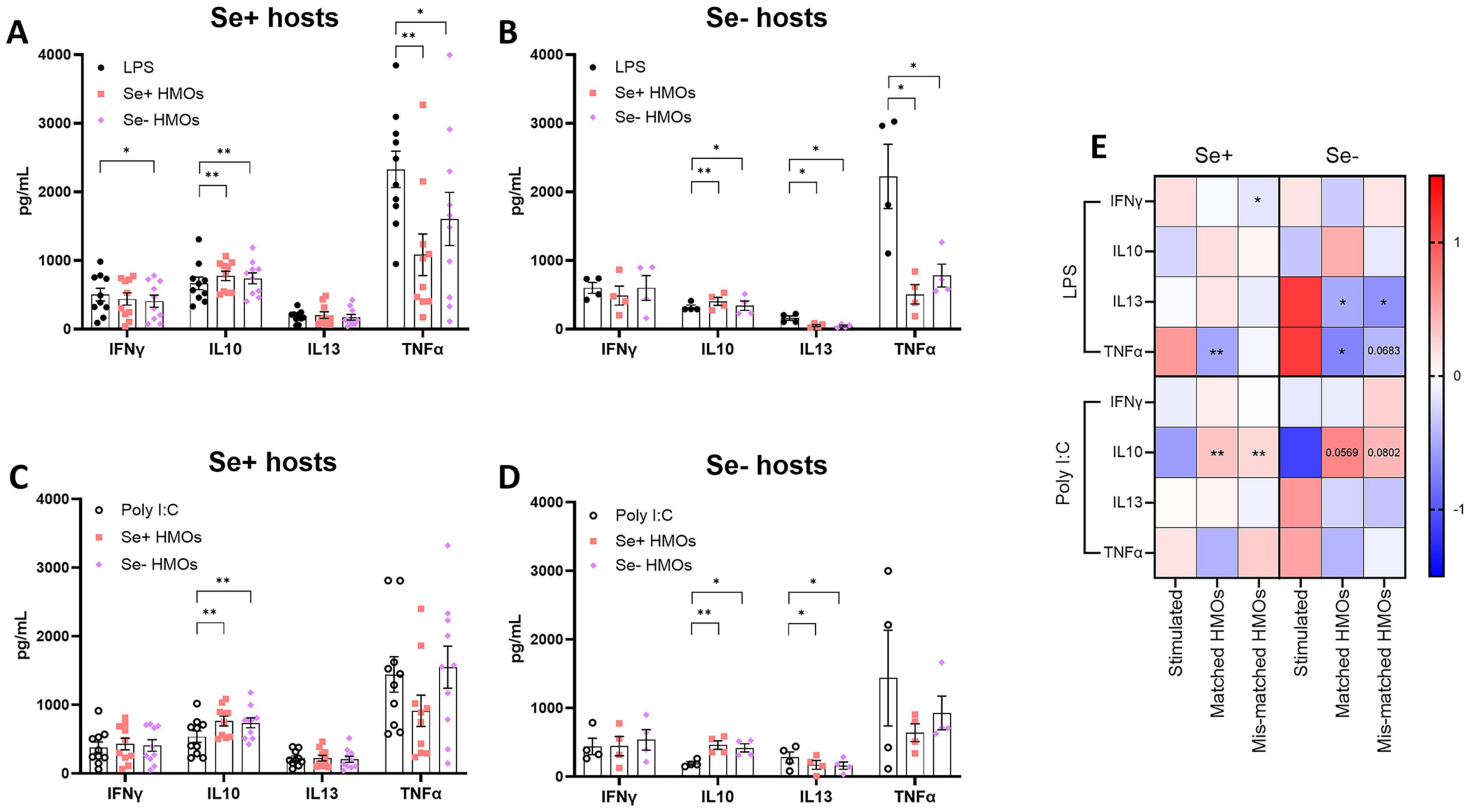
Immunomodulatory effects of pooled Se+ or Se− HMOs on cytokine secretion in LPS and Poly I:C stimulated Se+ and Se− PBMCs. PBMCs of 14 independent healthy donors were cultured for 48 h (with αCD3/CD28 stimulation) in the presence of 0.1 w/v% Se+ HMOs or Se− HMOs. During the final 24 h LPS or Poly I:C were added as additional stimulus. Secretion of cytokines was determined in **(A)** Se+ PBMCs exposed to pooled Se+ or Se− HMOs for 48 h and stimulated with LPS during the final 24 h, **(B)** Se− PBMCs exposed to pooled Se+ or Se− HMOs for 48 h and stimulated with LPS during the final 24 h, **(C)** Se+ PBMCs exposed to pooled Se+ or Se− HMOs for 48 h and stimulated with Poly I:C during the final 24 h, and **(D)** Se− PBMCs exposed to pooled Se+ or Se− HMOs for 48 h and stimulated with Poly I:C during the final 24 h. **(E)** *Z*-score of cytokine secretion are displayed in a heatmap to provide an integrated comparison across cytokines with differing dynamic ranges. **(A–D)** Data (*n* = 14 healthy PBMC donors) is analyzed by One-Way or **(E)** Two-Way ANOVA and Dunnett’s *post hoc* test. Data is displayed as mean ± SEM (* *p* < 0.05; **p < 0.01).

To investigate bacterial and viral immune responses, PBMCs were additionally exposed to LPS or Poly I:C, respectively, during the last 24 h of the 48 h incubation with αCD3/CD28 and HMOs. In Se+ PBMCs, cytokine secretion upon LPS stimulation and HMO incubation was affected as compared to Se− PBMCs (Figure 4A). Specifically, IFNγ secretion was decreased upon Se− HMO incubation, while IL10 secretion was enhanced by both Se+ and Se− HMOs. Additionally, both Se+ and Se− HMOs exposure resulted in lower levels of TNFα secretion in response to LPS. A different immunomodulatory response was observed in Se− PBMCs in response to LPS stimulation and HMOs incubation (Figure 4B). IL13 and TNFα levels were decreased irrespective of HMOs incubation while stimulated with LPS (Figures 4A,B).

Both Se+ as well as Se− PBMCs (Figures 4C,D) secreted increased levels of IL10 in response to both Se+ or Se− HMOs when stimulated with Poly I:C. Furthermore, only in Se− PBMCs (Figure 4D), a decrease of IL13 secretion was observed in the presence of HMOs when stimulated with Poly I:C.

Calculated z-scores revealed a specific Secretor status-matched effect on TNFα secretion in response to LPS; where significant reduction in TNFα secretion was only observed when PBMCs were incubated with Secretor-matched HMOs (Figure 4E). Additionally, previously observed patterns - increased IL10 secretion in Se+ PBMCs and decreased IL13 secretion in Se− PBMCs in response to HMO exposure remained consistent. These data show that upon bacterial as well as viral trigger, the cytokine response is mainly shaped by the PBMC Secretor status over the HMOs.

## 4 Discussion

This study provides novel insights into the immunomodulatory effects of HMOs in the context of Secretor status by demonstrating that PBMCs from Se+ and Se− individuals respond differentially to Se+ and Se− HMOs, as well as to bacterial and viral immune triggers. Our findings support the hypothesis that not only maternal Secretor status and associated HMO composition in human milk is relevant for immune cell responsiveness, but also host’s Secretor status. This potentially contributes to individual differences in immune development and disease susceptibility (7, 10, 11, 14).

We observed that FUT2 protein expression alone did not correlate with cytokine secretion in unstimulated PBMCs (Figures 2A–D), suggesting that basal FUT2 expression is not a direct determinant of immune activity in the absence of stimulation. Since we could only base Secretor status on FUT2 protein levels measured by ELISA, this method served as a practical but suboptimal proxy for Secretor status. This limitation should be considered when interpreting the data. However, upon stimulation, Se+ PBMCs exhibited a more robust IL10 and IL13 response as compared to Se− PBMCs (Supplementary Figure 1), indicating that FUT2 expression may prime immune cells for a more regulatory and type 2-skewed response under activating conditions. This aligns with previous findings that Se+ individuals may have a distinct immune profile compared to Se− individuals (10, 11, 21). Additionally, the observed stimulation-dependent differences between Se+ and Se− PBMCs indicate that our suboptimal FUT2 expression measurements capture biologically relevant variation.

Our data shows that both Se+ and Se− HMOs can enhance IL10 secretion in Se+ PBMCs, while this effect was less pronounced in Se− PBMCs. Interestingly, the individual HMOs, 2’FL and 3FL, did not replicate the effects of pooled HMO mixtures, underscoring the importance of studying complete HMO profiles in addition to isolated components. This supports the notion that the immunomodulatory capacity of HMOs arises from their structural diversity and synergistic interactions (22, 23).

IL13 secretion was particularly sensitive to host Secretor status, with Se− PBMCs showing a consistent decrease in IL13 levels despite Secretor profile of HMOs. This indicates that Se− individuals may have a dampened type 2 immune response after exposure to HMOs, which may have implications for allergic disease susceptibility. Notably, this effect was observed also under viral and bacterial stimulation, indicating that HMO-mediated modulation of IL13 is robust across different immune contexts. To the best of our knowledge, no direct correlation has been established between Secretor status and the development of allergic diseases (18, 24), although associations between Secretor status and susceptibility to, as well as severity of, viral infections have been reported (11).

The interplay between HMOs and immune triggers revealed nuanced effects dependent on Secretor status. For instance, Se+ PBMCs showed reduced TNFα and IFNγ secretion in response to LPS when incubated with Se− HMOs, while IL10 was consistently elevated in the same conditions. In contrast, Se− PBMCs exhibited a broader suppression of pro-inflammatory cytokines (IL13 and TNFα) regardless of the added HMO type. These findings suggest that HMOs may fine-tune immune responses in a context- and genotype-specific manner, potentially influencing susceptibility to infections and inflammatory diseases (25).

The observation that TNFα secretion was stronger reduced only when PBMCs were incubated with Secretor-matched HMOs is particularly intriguing. This match-specific effect implies a potential evolutionary advantage in maternal–infant Secretor status concordance, possibly optimizing immune training during early life. However, recently it was described that in Secretor-matched mother-infant pairs the infant is at intermediate risk of development of celiac disease and gastroenteritis. Interestingly, non-Secretor infants with a Secretor mother show the lowest risk of developing these conditions, while the highest risk was found in Secretor infants with a non-Secretor mother (19). This demonstrates that Secretor status-matched mother-infant pairs may not universally confer immunological advantage; rather, the interplay between maternal and infant Secretor status appears to shape disease susceptibility in a context-dependent manner (11), potentially reflecting a trade-off between immune training and pathogen defense during early life.

This study is limited by its *in vitro* design, which cannot fully replicate the complex environment of the infant gut or the developing immune system. While adult PBMCs offer practical advantages, they may not accurately reflect neonatal immune responses. Future studies should consider using cord blood or neonatal immune cells to enhance physiological relevance to study the immunomodulatory effects of HMOs. Additionally, classifying Secretor status based solely on FUT2 protein expression does not capture the full spectrum of genetic and metabolic variation influencing HMO profiles (12). A practical limitation of our dataset is the relatively low number of Se− donors, which reflects their lower prevalence in the general population and restricts the statistical power for this subgroup, although the observed differences remain consistent across individuals. Building on this exploratory work, future studies with larger and more balanced donor cohorts will be essential to validate and mechanistically extend these findings. Such mechanistic studies are needed to uncover the specific receptors and signaling pathways through which HMOs influence immune function. Gaining this understanding could reveal how HMOs shape immune cell behavior and contribute to immune development in early life. Additionally, the role of the gut microbiome in mediating HMO-immune interactions remains insufficiently understood. Exploring this relationship could clarify the potential contribution of microbial composition in HMO-driven immune modulation. Ultimately, translating these findings into clinical practice will require studies that assess how individual HMOs or HMO profiles affect infant health outcomes, potentially guiding the development of targeted and personalized nutritional interventions.

Our findings contribute to a growing body of evidence that HMOs are not merely prebiotics, but active modulators of immune function. The differential responses observed between Se+ and Se− PBMCs highlight the importance of considering infant genotype in studies of HMO function and maybe even in the design of HMO-supplemented infant formulas. Moreover, the Secretor status match between mother and infant may represent a previously underappreciated factor in shaping immune development. Future studies should explore the *in vivo* relevance of these findings, particularly in longitudinal cohorts tracking immune outcomes and disease incidence in relation to maternal–infant Secretor status. Additionally, mechanistic studies are needed to reveal the receptors and signaling pathways through which HMOs exert their effects on immune cells.

## Data Availability

The raw data supporting the conclusions of this article will be made available by the authors, without undue reservation.
