# Supplementary material for: Secretor status-dependent modulation of in vitro immune responses by human milk oligosaccharides
Source: Front Nutr. 2026 Jun 12;13:1791158. doi: 10.3389/fnut.2026.1791158 (PMC13348069; doi:10.3389/fnut.2026.1791158)
Supplement: Supplementary file 1 [file Supplementary_file_1.DOCX]

Supplementary Material

1. **Supplemental Figures**


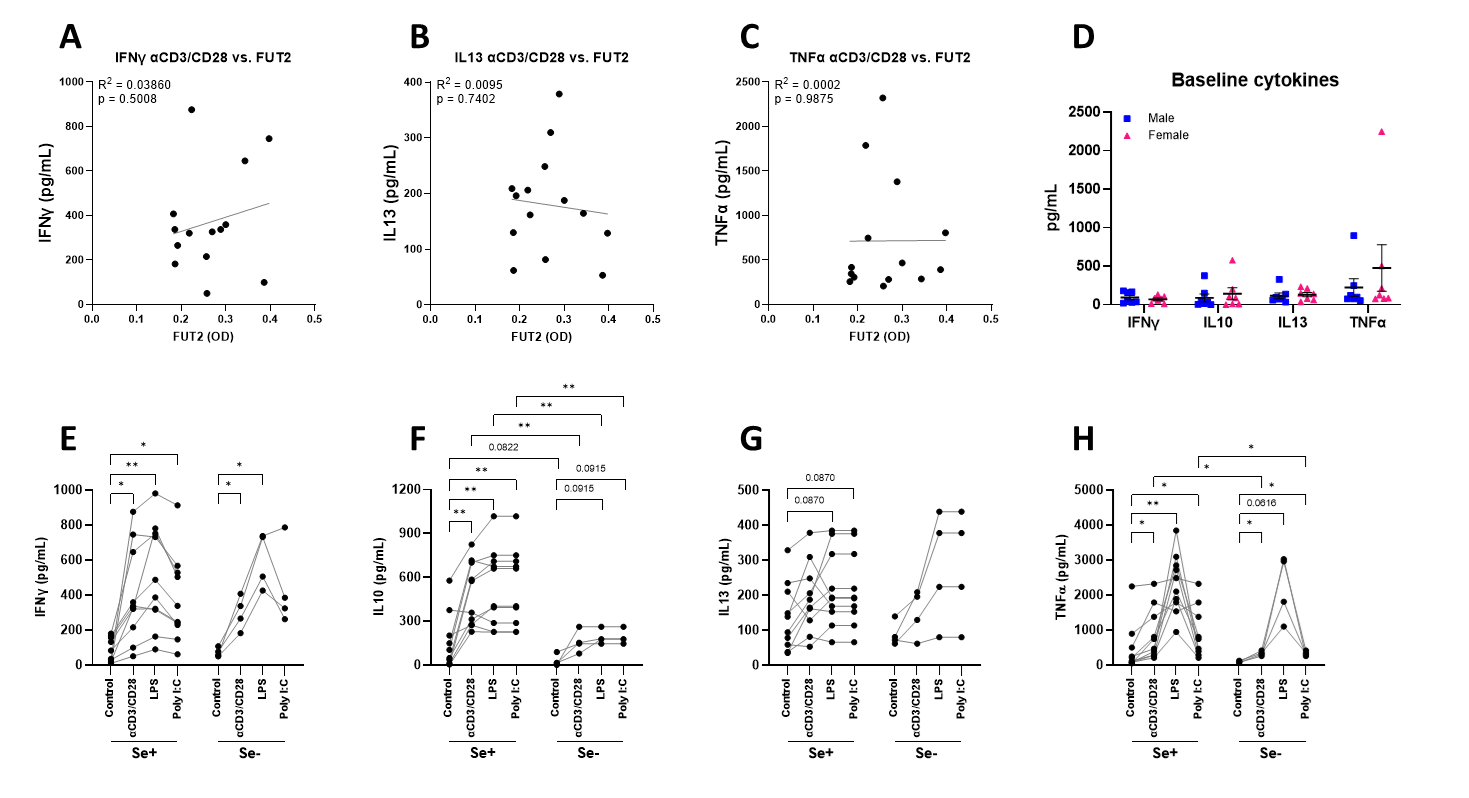


Supplemental Figure 1. No correlations are observed between A) IFNγ, B) IL13 and C) TNFα secretion after 48h αCD3/CD28 stimulation of PBMCs and FUT2 protein expression. D) Differences in cytokine secretion between PBMC donors are not explained by sex. Responses to all stimulations (αCD3/CD28, LPS and Poly I:C) of Se+ and Se- PBMCs are displayed. Although no significant differences between Se+ and Se- PBMCs were observed for E) IFNγ or G) IL13 secretion upon stimulation, Se+ PBMCs secreted significantly higher concentrations of F) IL10 and H) TNFα upon LPS or Poly I:C stimulation compared to Se- PBMCs. Data (n=14 healthy PBMC donors) is analyzed by Pearson correlation test and a simple linear regression. Cytokine secretion of unstimulated PBMCs were analyzed by Two-Way ANOVA with Bonferroni’s post hoc test and displayed as mean ± SEM (* p<0,05, **p<0,01).
